# The Midwives Act

**Published:** 1904-03-12

**Authors:** 


### The Hospital.

A Journal of

The Medical Sciences and Hospital Administration.

Vol. XXXV.—No. 911. MARCH 12, 1904.

### The Midwives Act.

The Wandsworth division of the Metropolitan Branch of the British Medical Association recently appointed a special committee to consider and report upon the Midwives Act of 1902, and the regulations of the Central Midwives Board; and the report of this committee is now before us. It embraces a considerable variety of subjects, but is clearly marked, from beginning to end, by a definite tone of hostility to the Act, and to the midwives rendered legitimate by it, a tone which, from the point of view of public duty, appears to us to furnish matter for regret. The first paragraph declares that it has been found exceedingly difficult to decide on any plan for common action. With regard to rendering assistance to the patients of midwives, it was "realised to be injudicious" to "advise all to decline to have anything to do with these midwives," and it was found necessary to "arrange some way by which it would be known who the practitioners might be who did not care to be called on at all," and the suggestion for meeting the difficulty is that the local supervising authority, in this case the London County Council, be asked to circularise the profession within its district, and then to issue a list of the practitioners who would be willing to go to the assistance of a midwife in case of need, and to supply this list to the midwives within their area. It is of course conceivable that there may be, in any area, medical practitioners who, for various reasons, do not desire to attend midwifery at all, and do not regard such attendance as forming any part of their ordinary occupations; and it is manifest that such men ought not to be called upon. But we do not think that any general practitioner, who attends his own patients during labour, would be justified in refusing to attend a case of danger or urgency to which he was called unexpectedly, whether a midwife had been in charge or not; while it is obvious that the presence of a midwife would afford a certain degree of security against such a call being made upon insufficient grounds, as well as an adequate reason for leaving the patient as soon as the absence of real emergency had been ascertained. It is not only that medical men are in some respects rightly looked upon as public servants, receiving certain privileges, *e.g.* exemption from jury service, on this ground, but also that they lie under the common obligation of all citizens to obey the law, and to obey it in the spirit as well as in the letter, and in such a way as to produce all the advantages which it is calculated to afford. If they disapprove of it there are legitimate methods of striving for its amendment. For any body of practitioners in a district to declare that, because they do not approve of the Act, they will refuse their help to lying-in women upon whom midwives have been in attendance seems to us a proceeding which it would be very difficult to justify; and we have only to suppose such a refusal to be unanimous in order to realise that it might easily lead to the unnecessary sacrifice of both maternal and infantile life. We think that a lying-in woman in peril should be able to obtain the assistance of the nearest medical practitioner; and we do not envy his feelings if his refusal to attend, or if the foreknowledge that he would not attend if sent for, were to entail a fatal issue either to the mother or to the child.

The other features of the report are comparatively unimportant. There is a recommendation which we think unfortunate, to the effect that medical men should refuse to give certificates of good moral character to candidates for midwifery licenses, and should leave such certificates to be procured from the "clerical profession;" while at the same time it is proposed that the Central Council of the Association should call the attention of all ministers of religion in England and Wales to the importance of their not certifying from *personal* knowledge unless they possess it. This is to imply a serious charge of probable culpable carelessness on the one hand, and to suggest that the culpably careless people may properly give certificates of moral character on the other. The report further suggests that the Central Midwives Board should, by some "regulations," the nature of which is not explained, "prevent" certified midwives from "touting, advertising, or otherwise unfairly competing with registered medical practitioners," a confession of weakness which we can only regard as pitiable. In fact, the whole report contemplates, and its recommendations would do much to intensify or to create, a relation of antagonism between medical men and midwives which would be equally injurious to the profession and to the community.

The Central Midwives Board, as we reported last week, has in the meanwhile approached the Privy Council with a view to obtaining an amendment of the Act to provide for the payment of legally qualified medical practitioners when called in by midwives in difficult cases. In a general way, we presume, such provision exists, in the liability either of the Poor Law authorities or of the husband or parent of the patient; but there may, of course, be cases in which the husband, although not a pauper, may be too poor to pay a proper fee, and, in these, there can be no doubt that some public fund ought to be available.

